# Structural, Evolutionary, and Functional Analysis of the Protein O-Mannosyltransferase Family in Pathogenic Fungi

**DOI:** 10.3390/jof7050328

**Published:** 2021-04-23

**Authors:** María Dolores Pejenaute-Ochoa, Carlos Santana-Molina, Damien P. Devos, José Ignacio Ibeas, Alfonso Fernández-Álvarez

**Affiliations:** Andalusian Center for Developmental Biology (Pablo de Olavide University/Consejo Superior de Investigaciones Científicas/Junta de Andalucía), 41013 Sevilla, Spain; lolapejenaute@hotmail.com (M.D.P.-O.); csantmol@gmail.com (C.S.-M.); damienpdevos@gmail.com (D.P.D.); joibecor@upo.es (J.I.I.)

**Keywords:** Pmt, *Ustilago maydis*, glycosylation, appressorium, pathogens

## Abstract

Protein O-mannosyltransferases (Pmts) comprise a group of proteins that add mannoses to substrate proteins at the endoplasmic reticulum. This post-translational modification is important for the faithful transfer of nascent glycoproteins throughout the secretory pathway. Most fungi genomes encode three O-mannosyltransferases, usually named Pmt1, Pmt2, and Pmt4. In pathogenic fungi, Pmts, especially Pmt4, are key factors for virulence. Although the importance of Pmts for fungal pathogenesis is well established in a wide range of pathogens, questions remain regarding certain features of Pmts. For example, why does the single deletion of each *pmt* gene have an asymmetrical impact on host colonization? Here, we analyse the origin of Pmts in fungi and review the most important phenotypes associated with Pmt mutants in pathogenic fungi. Hence, we highlight the enormous relevance of these glycotransferases for fungal pathogenic development.

## 1. Introduction

Glycosylation is a post-translational protein modification in which specific sugar donor molecules are synthesized, added to specific amino acids in target proteins, and processed during their transfer throughout the secretory pathway [1]. Unfaithful protein glycosylation frequently hinders correct protein folding and stability and consequently compromises protein function [2]. As glycoproteins are involved in many biological processes [3], glycosylation is essential for early embryonic development [4,5]. Genetic defects in the formation of glycoconjugates cause muscular, developmental, and neurological disorders [6,7,8], generate abnormal inflammatory responses [9], and promote cancer cell metastasis [10]. Hence, the study of the complexity, heterogeneity, and relevance of the complete catalogue of glycoconjugates, called the glycome, is a well-established field of biomedical research [4]. 

Glycosylation is broadly conserved in evolution and is present in the three domains: Eukarya, Bacteria, and Archaea. However, the type of sugar attached and, most importantly, the conformation of the sugar molecules is highly variable across kingdom subdivisions [11,12,13]. Protein glycosylation in pathogenic fungi is particularly relevant due to its connection with virulence; many virulence factors are likely to be glycoproteins [3,14]. For example, crucial elements for infection have been identified in the glycosylation pathways of the smut fungus *Ustilago maydis* [15,16,17], a maize pathogen. These key factors are all conserved in other smut fungi but not in their hosts. This discovery has contributed to the development of protein glycosylation studies as an emerging field in biotechnology. 

## 2. Protein Glycosylation in Fungi

**Figure 1:**
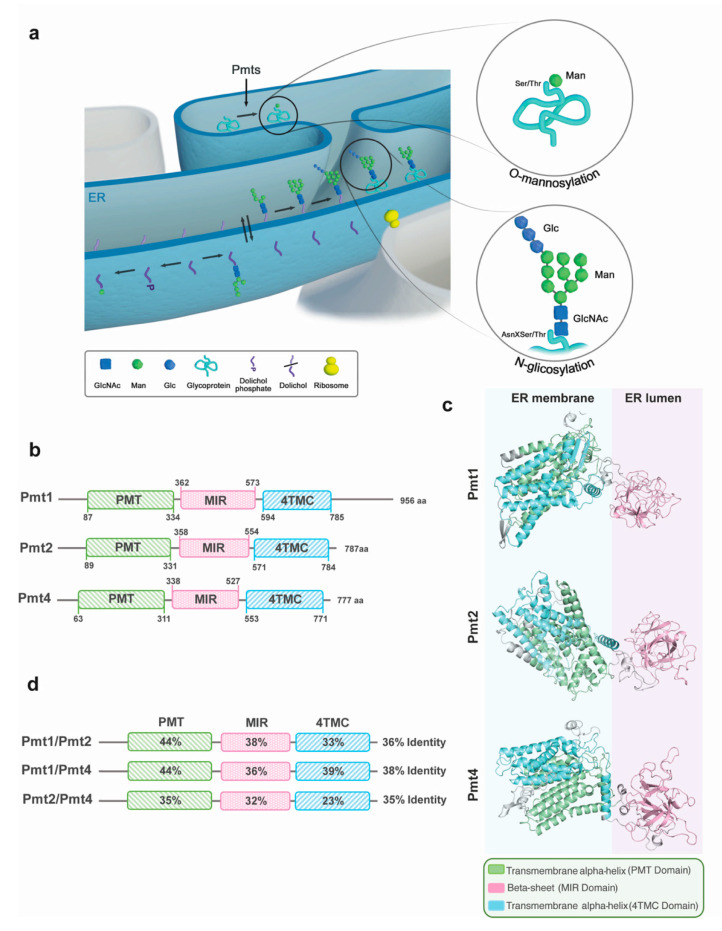
The Pmt protein family in the corn smut fungus *Ustilago maydis*. (**a**) Schematic of N-glycosylation and O-mannosylation pathways at the endoplasmic reticulum (ER) in fungi. N-glycosylation (below); the oligosaccharide core is assembled on the cytoplasmatic side of the ER, then translocated into the ER where four mannose and three glucose residues are added to the structure. A circle amplifies the polysaccharide structure which is transferred to an Asn residue of the polypeptide in the sequence Asn-X-Ser/Thr. O-mannosylation (above); protein O-mannosyltransferases (Pmts) add only one mannose to the Ser/Thr residues of the target polypeptide. Nascent glycoproteins undergo a later modification at the ER and Golgi apparatus. (**b**) Schematic structure of *U. maydis* Pmts (UmPmts). Pmt1 (Umag_11220), Pmt2 (Umag_10749), and Pmt4 (Umag_05433) conserve the three canonical Pmt domains: PMT (green), MIR (pink), and 4TMC (blue). The domains were identified using the Pfam database. (**c**) Predicted topology of UmPmt family according to [42]. UmPmt proteins harbour seven transmembrane alpha helices in the PMT domain (green), except Pmt1 which shows six helices. The MIR harbours beta sheet structures oriented into the ER lumen. The 4TMC is composed of four transmembrane alpha helices. PDB structures were obtained using Phyre and represented using PyMol. (**d**) Similarity comparison between UmPmts. Alignments and percentages of identity were obtained using Clustal Omega.

Based on studies over the past three decades in the budding yeast *Saccharomyces cerevisiae*, the pioneer model organism for the characterization of protein glycosylation pathways, two major types of glycosylation can be defined in fungi: N-glycosylation and O-mannosylation (Figure 1a). Both take place at the endoplasmic reticulum (ER) and Golgi apparatus [18,19]. 

### 2.1. N-Glycosylation 

In N-glycosylation, GlcNAc_2_Man_9_Glc_3_—an oligosaccharide comprising two N-acetylglucosamines (GlcNAc), nine mannoses (Man), and three glucoses (Glc)—is attached to the site-chain nitrogen atom of an acceptor Asn residue in the sequence Asn-*x*-Ser/Thr, where *x* can be any amino acid except proline [20,21,22,23]. Production of N-glycoproteins occurs in three sequential stages: synthesis of a lipid-linked-oligosaccharide, transfer of the oligosaccharide to the protein, and processing of the glycosidic structure. The first stage of the N-glycosylation pathway occurs on both the cytosolic and lumenal sides of the ER membrane. On the cytosolic side, a dolichol monophosphate (Dol-P) molecule acts as a lipid carrier to link the oligosaccharide (GlcNAc_2_Man_5_) to the ER membrane. GlcNAc_2_Man_5_ is then moved to the lumenal side of the ER by a flippase-like protein. Core oligosaccharide synthesis finishes with the addition of four more mannoses and three glucoses. The oligosaccharyltransferase complex enables the attachment of this sugar core to Asn residues in the consensus sequence of nascent proteins. Finally, the sugar core undergoes a sugar trimming process in which the three glucoses are sequentially removed by glucosidases I and II (Figure 1a) [24,25,26,27,28,29,30]. 

### 2.2. O-Mannosylation and the Pmt Family

Protein O-glycosylation is characterized by the addition of oligosaccharides to an OH group of Ser or Thr amino acids without known consensus sequence [31]. As in N-glycosylation, O-glycosylation is conserved from bacteria to humans [18,20,32,33]. However, the structure of the oligosaccharide attached to the target protein varies [21].

O-mannosylation is the most common type of O-glycosylation in fungi that is characterized, in this division, by an oligosaccharide composed mainly of mannoses [34,35]. Protein O-mannosyltransferases (Pmts) mediate the transfer of mannoses from Dol-P-activated mannose (Dol-P-Man) to target proteins in the ER (Figure 1a) [36]. The Pmt protein family is widely conserved as its members are crucial for many biological functions. In pathogenic fungi, Pmts are key to virulence due to their roles in maintaining cell wall integrity and secretion of fungal effectors [14,16,37,38,39,40]. In plant pathogenic fungi, the Pmt family consists of three members: Pmt1, Pmt2, and Pmt4. Despite the relevance of Pmts to fungal virulence, there are still open questions about their role in infection. For instance, the fact that deleting each member produces a phenotype that differs in pathogenesis suggests that Pmts have a range of substrates, most of which are still unidentified [14]. Significantly, the conservation of the crucial role of Pmt4 in fungal plant pathogenesis [16,38,40,41], together with the absence of Pmt4 orthologues in its hosts, makes Pmt4 an excellent candidate for targeting by antifungal drugs. Here, we summarize much of what is known about the structure, evolution, and function of Pmt family members. We focus on their connections to fungal pathogenesis and, in particular, their known role in *U. maydis*, one of the model organisms in which the function of Pmts and their substrates have been explored more widely.

*U. maydis* is a biotrophic fungus that combines a nonpathogenic cell cycle, in which it divides by budding asexually, with a pathogenic stage that starts with the fusion of two sexually compatible strains [43]. Once cells fuse on the plant surface, plant physicochemical signals trigger the formation of a filamentous pathogenic hyphae which accesses plant tissues by developing the appressorium—a morphogenetic structure that mediates plant penetration [44]. Inside the plant, the fungal hyphae expand in size and number, inducing the formation of plant tumours containing *U. maydis* spores [45]. The role of Pmts in *U. maydis* virulence has been explored in recent decades. While Pmt1 seems not to play a relevant role in infection, Pmt2 is required for cell survival and Pmt4 is essential for appressorium formation and penetration. Remarkably, several Pmt4 substrates were identified as responsible for the virulence defects caused by mutation of *pmt4* [14,16]. 

## 3. The Structure of Pmts

Pmts are multispanning ER membrane proteins that contain three domains. Two ER transmembrane α-helix regions: the PMT (protein O-mannosyltransferases) and 4TMC (4 transmembrane domains in the C-terminal region) domains are located at the N- and C-termini, respectively. The third domain is the central MIR domain, so named because it is common to mannosyltransferases, inositol triphosphate receptors, and ryanodine receptors [46], and has a β-trefoil fold [47,48]. The MIR domain, positioned at the ER lumen, interacts directly with the substrates [49] and thus might confer target specificity to Pmt proteins. These three domains, and their predicted 3D conformation, are conserved in *U. maydis* Pmts (Figure 1b,c). The three domains have a high percentage of sequence similarity in *U. maydis* Pmts, although the PMT domain is more similar between Pmts than the MIR and 4TMC domains are (Figure 1d). Moreover, Pmt1 is characterized by a longer C-terminal region harbouring a disordered domain that is conserved in the Pmt1 proteins from most fungi. The presence of this disordered domain differentiates Pmt1 from the other Pmts. On the other hand, Pmt1 in *U. maydis* contains only six TMs instead of seven—the canonical number of these O-glycosyltransferases [47]—which could indicate a slight divergence between Um Pmt1 compared with other Pmt1. 

Interestingly, Pmts act by forming dimers [50,51]. In particular, in budding yeast, Pmt1 interacts with Pmt2, and Pmt4 is able to interact with itself [50]. However, the fact that the loss of Pmt1 and Pmt2 has a different impact on cell cycle progression—as observed in a wide variety of species (Table 1) including *U. maydis* [16]—suggests that Pmt1 and Pmt2 might be active as monomers, interact with each other, or even with Pmt4, as observed in *Aspergillus nidulans* Pmts [52]. 

## 4. Evolution of the Pmt Protein Family

### 4.1. Bacterial and Eukaryotic Contributions to the Origin and Diversification of Pmts in Opisthokonts

**Figure 2:**
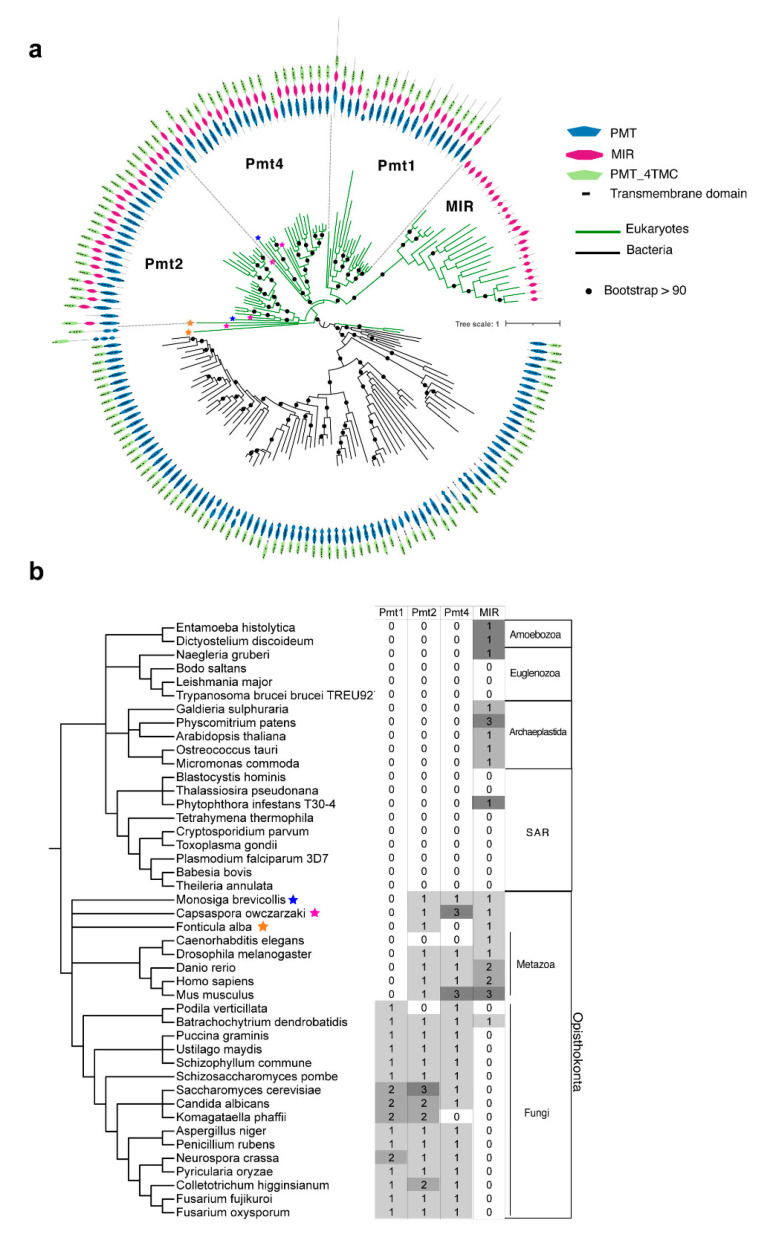
Evolutionary analyses of Pmt proteins. (**a**) Phylogeny and domain architecture of the closest homologs to Pmt proteins. This phylogeny represents the general features of the closest homologs to Pmt and is not for inferring the order of appearance of these protein families. Stars indicate early-branching opisthokonts. The tree was artificially rooted at the split of Pmt with and without MIR domains. An extended version of this reconstruction is provided in Figure S1. (**b**) Distribution of different Pmt proteins was identified in the phylogenetic reconstructions. The taxonomic tree was obtained from NCBI taxonomy tools (https://www.ncbi.nlm.nih.gov/taxonomy, accessed on 21 April 2021).

The closest homologous proteins to the Pmt protein family in pathogenic fungi are the eukaryotic Pmt1, Pmt2, and Pmt4; the prokaryotic mannosyltransferases that lack the MIR domain; and the isolated MIR domain found in distant eukaryotes (Figure 2a,b). In prokaryotes, archaea do not contain Pmts, and bacteria have Pmts with well-conserved PMT and 4TMC domains but without the crucial MIR domain (Figure 2a). In fact, O-mannosylation was commonly considered to be specific to eukaryotes but since then has been shown to be present in prokaryotes [13]. In particular, O-mannosylation seems to be well conserved across actinomycetes, as *Corynebacterium glutamicum* [62], *Mycobacterium tuberculosis* [63], and *Streptomyces coelicolor* [64] contain putative PMT-domain-containing transferases showing structural similarity to eukaryotic protein mannosyltransferases but without the MIR domain. The roles of these proteins in glycosylation have been experimentally validated: deletion in *C. glutamicum* causes a complete loss of glycosylation of the secreted proteins [62]; in *S. coelicolor*, these proteins are necessary for glycosylation of the phosphate-binding protein PstS [64]; and *M. tuberculosis* shows a strong attenuation of pathogenicity under deletion of these ancestral mannosyltransferases [63]. 

The monophyletic behaviour of Pmt sequences in phylogenetic reconstructions (Figure 2a) suggests that eukaryotic Pmt had a single origin and diversified by gene duplication. Orthologues of Pmt2 and Pmt4 are found in metazoa, fungi, and early-branching opisthokonts such as *Monosiga brevicollis*, *Capsaspora owczarzaki*, and *Fonticula alba* (Figure 2a,b and Figure S1), suggesting that their origin might precede the diversification of opisthokonts. By contrast, Pmt1 is found specifically in fungi, where it is well-conserved. We found other Pmt1-like sequences in *F. alba* and *C. owczarzaki* that branch basally to the whole eukaryotic Pmt family and have a divergent MIR domain (Figure 2a). These sequences provoked instability in different phylogenetic reconstructions (Figures S1 and S2); therefore, we suspect that the locations of both sequences represent well-known phylogenetic long-branch attraction artefacts which makes it difficult to infer the actual ancestrality of these sequences. Therefore, we conclude that, in contrast to Pmt2 and Pmt4 originating early in opisthokonts, Pmt1 originated later in the common ancestor of fungi. On the other hand, single MIR domains are found in diverse eukaryotes (opisthokonts, archaeplastida, and protists) forming a very distinct monophyletic group (Figure 2a), which suggests that the origin of MIR was established earlier in eukaryotic evolution, in contrast to Pmt proteins. Given the heterogeneity of the sequences and the low support at basal nodes in Figure 2a, it is difficult to infer a realistic order of appearance of these proteins from this reconstruction.

Hence, the close relationship between bacterial mannosyltransferase and Pmt proteins, together with the fact that the MIR domain is found on its own in eukaryotes (Figure 2a,b), suggests that the ancestor of opisthokonts gained the mannosyltransferase (PMT-4TMC) by lateral gene transfer (LGT) from bacteria. Mannosyltransferases without MIR domain (PMT-4TMC) were also found in other eukaryotes, for instance, in the red algae *Galdieria sulphuraria*. However, given the discrete distribution of these proteins in eukaryotes, we reckon that these cases represent independent LGT origins from bacteria. Once the opisthokont ancestor gained by LGT the PMT-4TMC from bacteria, the eukaryotic MIR domain was inserted. Later, this first ancestral eukaryotic PMT-MIR-4TMC would have duplicated in the ancestor of opisthokonts, leading to Pmt2 and Pmt4, forming a well-conserved protein family, despite some punctual losses as in *Caenorhabditis elegans* (Figure 2b). 

### 4.2. The Origin of Fungi-Specific Pmt1 Might Be Pmt4

**Figure 3:**
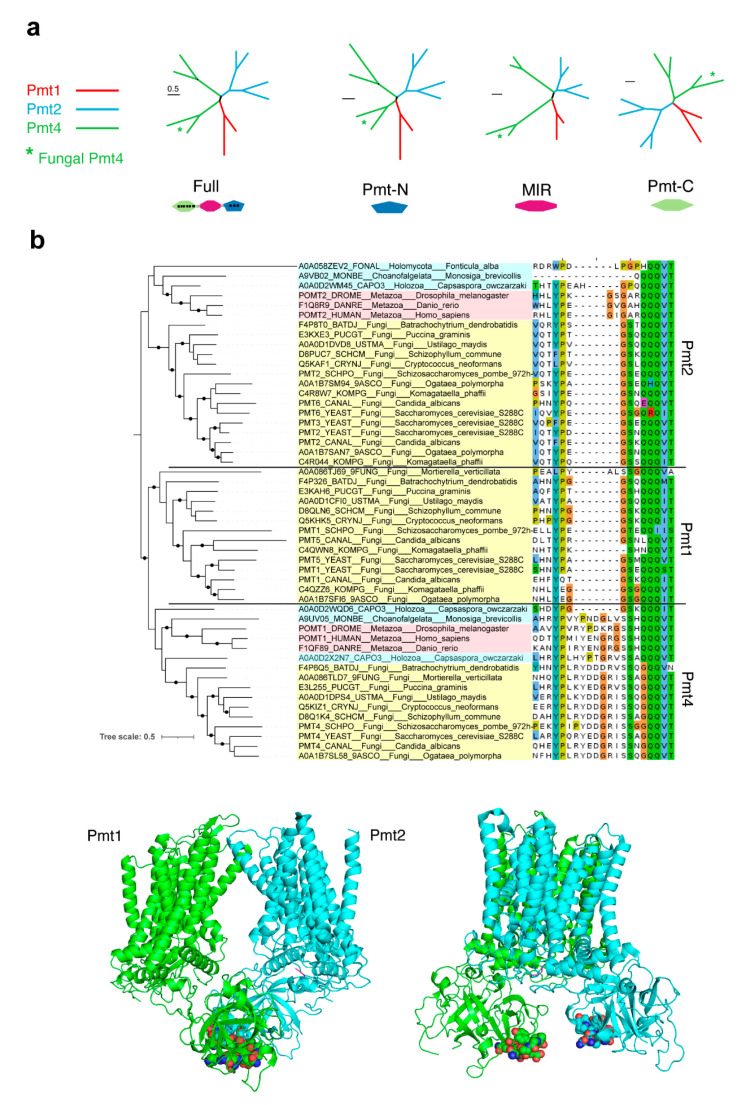
The origin of fungi-specific Pmt1 might be Pmt4. (**a**) Phylogenetic distances of the whole Pmt protein and single domains between the Pmt sequences of *Homo sapiens, Drosophila melanogaster, Saccharomyces cerevisiae*, and *Ustilago maydis*. (**b**) Phylogeny of eukaryotic Pmt proteins (**left**) and multiple sequence alignment of the region with an indel between the positions 387 and 388 (*U. maydis* Pmt2 positions) within the MIR domain, common in Pmt2 and Pmt1 (**right**). Labels were highlighted according to the taxonomy: blue, early branching opisthokonts; pink, metazoan; and yellow, fungi. Black circles at nodes indicate bootstraps higher than 90. A ribbon representation of the structure of the Pmt1 and Pmt2 dimer (PDB code 6p25) is shown below, with the residues surrounding the indel represented in spheres.

We further explored the possible origin of Pmt1 from a previously established ancestral form, Pmt2 or Pmt4. We investigated the relationships between the three Pmts from different species (*Homo sapiens*, *Drosophila melanogaster*, *S. cerevisiae*, and *U. maydis*) through the domains that compose them (Figure 3a). The phylogenetic distances between the whole protein and the single domains show that Pmt1 is more closely related to the fungal Pmt4 than to Pmt2. In addition, phylogenetic reconstruction of the whole Pmt protein with extended taxonomic sampling shows that Pmt1 and Pmt4 are slightly more similar to each other than to Pmt2 (phylogenetic tree in Figure 3b). These results suggest that Pmt4 might be the origin of Pmt1 in fungi. 

## 5. Variability in the Number of Pmt Family Members

### 5.1. The Pmt Family in Animals Consists of Two Members

Two O-mannosyltransferases, usually named POMT1 and POMT2 (protein O-mannosyltransferase 1 and 2), form the Pmt family in animals. The biological relevance of the POMTs has been experimentally validated in some animal species. For instance, in *D. melanogaster*, POMT1 and POMT2 are required for maintaining the integrity of larval muscles [65,66] and normal axonal connections of sensory neurons [67], while in humans, mutations in *POMT1* and *POMT2* lead to Walker–Warburg syndrome [68,69,70]. Therefore, the presence of the two members of the Pmt family is the most common situation in opisthokonts and indicates that two Pmts are enough to efficiently carry out faithful protein O-mannosylation in these types of eukaryotic cells. 

### 5.2. The Addition of a Third Pmt in Fungi

**Figure 4:**
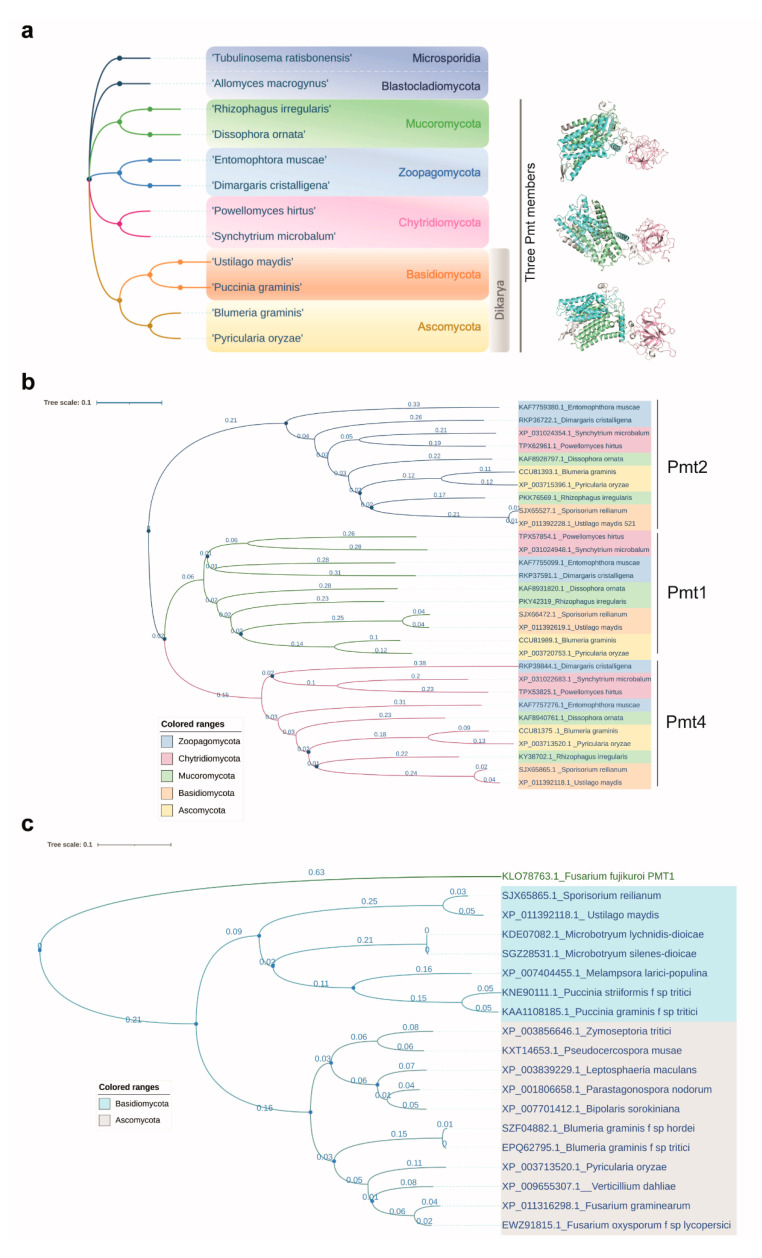
The Pmt family is commonly composed of three members in fungi. (**a**) Most fungal clades harbour three Pmts. The taxonomic tree was obtained using the NCBI taxonomy browser. The number of Pmts in each group was obtained using BlastP. (**b**) The species selected in (**a**) are represented in a phylogenetic tree. The blue bar represents an evolutionary distance of 0.1. Blue circles at nodes indicate bootstrap higher than 90. (**c**) Conservation of Pmt4 across plant pathogenic fungi. Pmt4 is conserved in ascomycota and basidiomycota clades. The grey bar represents an evolutionary distance of 0.1. Blue circles at nodes indicate bootstrap higher than 90. *Fusarium fujikuroi* Pmt1 was used as an outgroup protein. The alignments were obtained using MAFFT v7 and 100 bootstrap replicates with the “bootstrap (valid for NJ)” option. The phylogenetic trees were generated using Archaeopteryx.js and edited in iTOL.

In fungi, the Pmt family is commonly composed of three members. Ascomycota, basidiomycota, chytridiomycota, zoopagomycota, and the other main fungal divisions harbour three Pmt gene sequences (Figure 4a,b). Our search showed that the addition of an extra member in the Pmt family is specific to fungi, as early-branching opisthokonts and animals harbour only two Pmts in their genomes (Figure 2a,b). In some ascomycetes, the Pmt family has more members, likely due to genome duplication events. In the budding yeast *S. cerevisiae*, for example, the Pmt family consists of seven members, Pmt1–7 [71,72]. As the role of the Pmt family was first analysed in this model organism, Pmts that were later identified and characterized in other fungal models adopted the same naming convention: *U. maydis* Pmts are named Pmt1, Pmt2, and Pmt4, as they are the closest orthologues to *S. cerevisiae* Pmt1, Pmt2, and Pmt4, respectively [16]. The presence of more than three members in the Pmt family has also been observed in other model fungi such as the human pathogen *Candida albicans* and *Pichia pastoris* (also known as *Komagataella pastoris*, Figure 2b), in which five Pmts have been characterized [60,73]. However, this is not always the case in ascomycota: only three Pmts have been identified in *Schizosaccharomyces pombe* [74]. Despite the fact that *S. cerevisiae*, *C. albicans*, and *P. pastoris* contain more than three Pmts, these proteins can be grouped into three subfamilies—Pmt1, Pmt2, and Pmt4—since there is functional redundancy between members of each subfamily [36]. 

Hence, the addition of a third or more Pmts in fungi might be favoured by the importance of O-mannosylation for their lifestyle, which requires the secretion of a high number of proteins, most of which are glycoproteins. Our analysis of representative species from the main fungi clades confirmed the presence of three Pmt proteins across fungi divisions (Figure 4a). In smut fungi, which have three Pmts, pathogenic development requires the secretion and activity of many virulence factors, which might be safeguarded by extra O-mannosyltransferases.

## 6. The Role of Pmts in Fungal Pathogenesis: Pmt4 as the Key Protein Factor in Virulence

Although full deletion of the Pmt family leads to lethality in fungi, loss of Pmt1, Pmt2, or Pmt4 has different consequences. For example, in most fungi, Pmt2 is essential for viability, whereas Pmt1 and Pmt4 are dispensable for cell growth. The high sequence similarity between the Pmts suggests that crucial changes in amino acids might be responsible for substrate specificity and thus the phenotypes associated with *pmt1*, *pmt2*, and *pmt4* mutants. Next, we summarize the main phenotypes of each Pmt in pathogenic fungi and speculate about the possible proteins that could be linked to the phenotypes associated with each mutation.

### 6.1. Pmt1, the Most Dispensable of the Pmts

Our comparison of Pmts shows that Pmt1 is the extra Pmt family member in fungal divisions. In most fungi in which the Pmt family has been characterized, *pmt1* mutants do not show major virulence defects; although the absence of Pmt1 leads to some growth defects in model organisms such as *Botrytis cinerea*, *Fusarium oxysporum, C. albicans*, and *Magnaporthe oryzae*, mutant cells do not show important virulence defects, except for a defect in plant penetration observed in *B. cinerea* ∆*pmt1* cells [38,41,59,60] (Table 1). Similar results were found in *A. nidulans*: although some defects in growth were observed in *pmt1* mutants, the penetrance of these defects is lower compared with that of other *pmt* mutants and no major defects in virulence were observed [54]. In the case of the smut fungus *U. maydis*, loss of Pmt1 has no significant impact on pathogenic development, because ∆*pmt1* cells are able to colonize the host and induce tumours in maize [16]. By contrast, severe defects in virulence associated with the loss of Pmt1 have been reported in *Cryptococcus neoformans* [75] and *Beauveria bassiana* [57], although the penetrance of these defects is always less than those of *pmt2* and *pmt4* mutations. In *B. bassiana*, the C-terminal Pmt1 MIR domain is dispensable for virulence [58]. 

This evidence collectively suggests that Pmt1 is the family member least relevant to cell cycle progression and virulence. This is probably because in the absence of Pmt1, Pmt2 and Pmt4 are sufficient to perform O-mannosylation of most glycoproteins, similarly to how POMT1 and POMT2 do in animals. 

### 6.2. Pmt2 and Its Essential Role for Cell Viability

In most fungal pathogens in which the Pmt family has been characterized, *pmt2* is an essential gene for viability [16,53,57,60,75]. Some exceptions are *B. cinerea*, *A. nidulans, Penicillium digitatum*, and *M. oryzae*, where ∆*pmt2* cells show severe growth defects [54,56,59,61] (Table 1). The relevance of Pmt2 for viability is also conserved in important nonpathogenic models such as *S. pombe* and *P. pastoris* [73,74]. There are several possible explanations for the lethality of ∆*pmt2* cells: (i) most O-mannosylated proteins might be glycosylated by Pmt2; (ii) O-mannosylated proteins that are crucial for cell cycle progression could be Pmt2-specific target proteins; and (iii) Pmt2 might physically interact with other proteins essential for viability. This last possibility would explain why, if Pmt1 and Pmt2 form a heterodimer, the loss of *pmt1* does not affect viability, but the loss of *pmt2* does. Alternatively, dimer formation between Pmt2 and Pmt4, as observed in *A. nidulans* [52], or a Pmt2 monomer, or homodimer, similar to the homodimerization of Pmt4 [50], could efficiently control the O-mannosylation process in ∆*pmt1* cells. 

### 6.3. Pmt4, the Key Pmt Protein in Virulence 

Among the three fungal Pmt proteins, Pmt4 is the most interesting from the point of view of virulence. Although it is not essential for viability in most of the fungal models in which its role has been explored, Pmt4 is crucial for pathogenic development [16,38,40,41]. Pmt4 might thus be the Pmt protein that O-mannosylates more virulence-related glycoproteins than the other Pmts do. In this context, it has been observed in *Fusarium oxysporum* and *Trichoderma reesei* that Pmt4 specifically O-mannosylates membrane proteins [39,41] and soluble proteins in the secretory pathways, particularly, GPI-anchored proteins. By contrast, Pmt2 is more related to substrates implicated in cell wall synthesis [41]. Consistent with this idea, loss of Pmt4 does not lead to severe defects in cell wall integrity, although some of these phenotypes have been linked to failures in cell wall integrity under stress conditions, e.g., in *C. neoformans* [55] (Table 1). However, *pmt4* mutants are mainly characterized by a drastic reduction in their virulence capability.

In plant pathogens, Pmt4 is crucial for appressorium penetration. This phenotype has been described for *U. maydis*, *M. oryzae*, and *Metarhizium acridum* [16,38,40] (Table 1). The mechanisms of appressorium penetration differ in *M. oryzae* and *U. maydis*: *M. oryzae* penetration is facilitated by the generation of high turgor pressure, whereas *U. maydis* uses the appressorium to mark the penetration point, where the controlled secretion of hydrolytic enzymes degrades the plant cuticle [76]. This difference suggests that Pmt4 substrates required for appressorium penetration might be conserved among species.

In addition to its role in appressorium penetration, Pmt4 is important in other processes associated with virulence. For example, ∆*pmt4* cells in *M. oryzae*, *M. acridum, F. oxysporum*, and *A. fumigatus* show defects in cell wall integrity, in polarized hyphae growth, and in mycelial development [38,40,41,53]. Moreover, Pmt4 has roles after penetration during *U. maydis* and *M. oryzae* hyphal expansion inside plant tissues, which points to this Pmt being required to suppress plant defence responses [14,76,77].

These observations indicate that Pmt4 is the Pmt family member with the most specific role in virulence in a wide range of fungal plant pathogens. Its moderate role during cell cycle progression makes Pmt4 an attractive protein for understanding the mechanisms behind fungal virulence and for identifying the main secreted proteins that are important for their pathogenic development. Due to its conservation in pathogenic fungi (Figure 4b), its absence in its hosts, its specific role in virulence, and its crucial role in the formation of the appressorium—a critical stage at which the progression of plant infection and the loss of crops can be arrested—Pmt4 is a suitable target for the development of more specific antifungal treatments.

## 7. What Pmt4 Substrates Might Justify the Virulence Defects Observed in *pmt4* Mutants in *U. maydis*?

An in silico screening of 6787 proteins in *U. maydis* identified 64 proteins harbouring two of the most relevant features of Pmt4 substrates: at least one transmembrane domain and a longer region of 40 amino acids in which the proportion of Ser or Thr is greater than 40% [14,78]. Interestingly, in this search, we identified the signalling mucin Msb2 as a putative substrate of Pmt4. Msb2 is required for appressorium formation in *U. maydis* and *M. oryzae*, likely because of its important role in detecting the plant signals required for triggering appressoria development [79,80,81]. The fact that the Ser/Thr-rich region of Msb2 is required for Msb2 function in *U. maydis* and the epistatic relationships between *msb2* and *pmt4* suggest that the appressorium defects associated with the loss of Pmt4 might be a consequence of defective O-mannosylation of the signalling mucin receptor.

**Figure 5:**
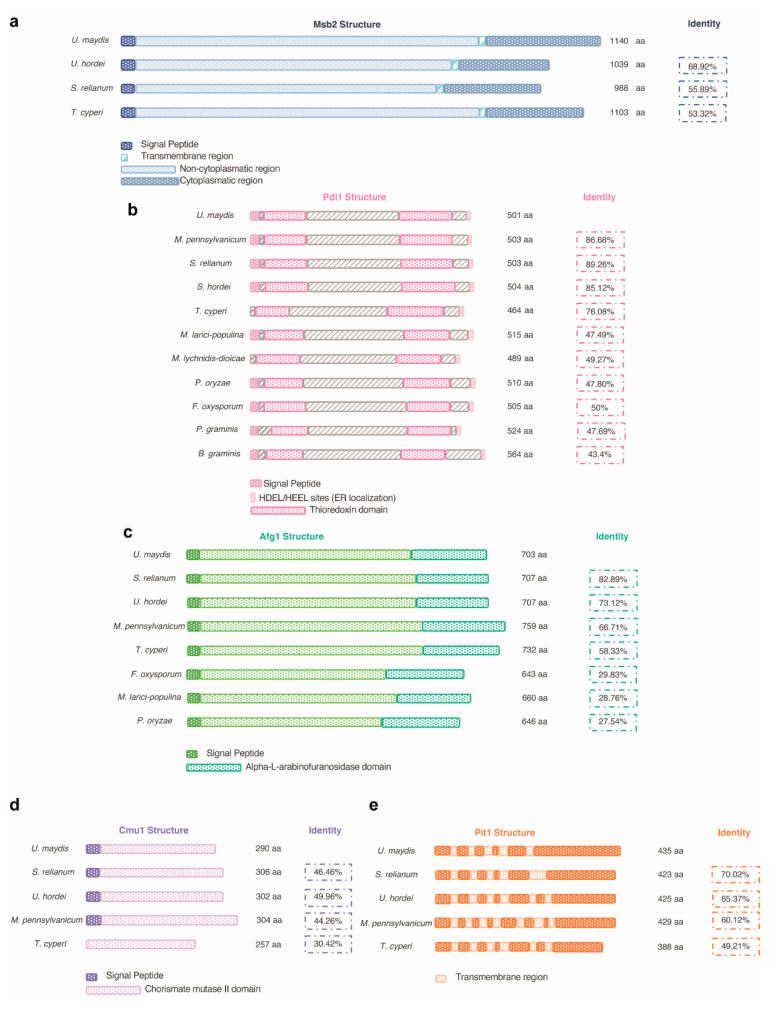
Schematic representation and conservation of Pmt4 putative targets across fungi. The percent identity of the protein was obtained using BlastP and is plotted to the right of each protein. Organisms used to study the conservation of Pmt4 putative targets were: *Ustilago hordei, Sporisorium relianum, Melanopsichium pennsylvanicum*, and *Testicularia cyperi (smut fungi); Melampsora larici-populina*, *Microbotryum lychnidis-dioicae*, and *Puccinia graminis* (basydiomycota); and *Pyricularia oryzae, Fusarium oxysporum, and Blumeria graminis* (ascomycota). *Ustilago maydis* sequence was used as template. Schematic structure of the proteins was obtained from InterPro. (**a**) Msb2 (UMAG_00480) presents a signal peptide and a transmembrane region with a cytoplasmatic and a noncytoplasmatic side. This protein and its structure are conserved in the smut fungi *U. hordei* (CCF54229.1), *S. relianum* (SJX60528.1), and *T. cyperi* (PWZ02537.1). (**b**) Pdi1 (protein disulfure isomerase, UMAG_10156) presents a signal peptide, two thioredoxin domains, and HDEL/HEEL ER localization sites. This protein and its structure are conserved in the smut fungi *M. pennsylvanicum* (CDI55397.1)*, U. hordei* (CCF48050.1)*, S. relianum* (CBQ71186.1), and *T. cyperi* (PWY98840.1). It is also conserved in the basidiomycota fungi *M. larici-populina* (XP_007411270.1)*, M. lychnidis-dioicae* (KDE03239.1), and *P. graminis* (KAA1097218.1) and in the ascomycota fungi *P. oryzae* (XP_003710672.1)*, F. oxysporum* (EGU89226.1)*,* and *B.graminis* (KAA1097218.1). (**c**) Afg1 (alpha-L-arabinofuranosidase I precursor, UMAG_01829) presents a signal peptide (SP) (except *T. Cyperi* and *M. larici-populina*) and an alpha-L-arabinofuranosidase domain. This protein and its structure are conserved in the smut fungi *S. relianum* (SJX61927.1)*, U. hordei* (CCF53267.1)*, M. pennsylvanicum* (CDI52700.1), and *T. cyperi* (PWY98448.1). It is also conserved in the basydiomycota fungus *M. larici-populina* (XP_007407432.1) and in *P. oryzae* (XP_003712124.1) and *F. oxysporum* (RKL11611.1). (**d**) Cmu1 (chorismate mutase I, UMAG_05731) presents a signal peptide, and a chorismate mutase domain. This protein and its structure are conserved in the smut fungi *S. relianum* (SJX65239.1)*, U. hordei* (CCF49464.1)*, M. pennsylvanicum* (CDI51551.1), and *T. cyperi* (PWZ01961.1). (**e**) Pit1 (UMAG_01374) is conserved in smut fungi and presents five transmembrane helices in *U. maydis* and *S. relianum* (SJX61460.1), six in *U. hordei* (CCF54347.1) and *T. cyperi* (PWZ03382.1), and three in *M. pennsylvanicum* (CDI52271.1).

In addition to showing defects in appressorium formation, ∆*pmt4* cells are unable to break the plant cuticle. In these cases, penetrance of this phenotype is even higher than the penetrance of the defects in appressorium formation; although some *pmt4* mutant hyphae can develop into an appressorium, they totally lose their ability to penetrate the plant cuticle [16]. The target(s) that might explain appressoria penetration defects are not yet known. By contrast, the role of Pmt4 inside the plant might be explained by the O-mannosylation of a range of important substrates; for instance, Pit1, Cmu1, and Afg1 are possible Pmt4 substrates required for full pathogenic development [81,82,83] and are conserved in pathogenic fungi (Figure 5). Recently, it was demonstrated that the protein disulfide isomerase Pdi1 shows altered electromobility in *pmt4* mutants [37]. Pdi1, located at the ER, assists glycoproteins in folding and disulfide bond formation. A large number of Pmt4 and Pdi1 substrates might support the role of Pmt4 in fungal proliferation inside plant tissues.

What feature of Pmt4 might confer the ability to specifically O-mannosylate these substrates but not be O-mannosylated by Pmt1? We hypothesized that a Pmt4-specific sequence might control specificity for substrates. To test this idea, we aligned the MIR domains of all Pmts and POMTs and identified the amino acid sequence LRYDDGRVS as conserved in the Pmt4s (POMT1s) but absent in Pmt1s and Pmt2s (Figure 3b). This part of the MIR domain, or any other Pmt4 region that differs slightly from the two other Pmts, might be implicated in Pmt4-specific recognition and therefore the glycosylation of different targets. This could explain the different phenotypes associated with the loss of Pmt1, Pmt2, or Pmt4. We are currently aiming to decipher the basis of this preferential glycosylation.

## 8. Conclusions

In this work, we summarize the main features of the structure, evolution, and function of the O-mannosyltransferases in fungi. In addressing important questions about the essential role of Pmts in pathogenic fungi, our evolutionary analyses suggest that Pmt2 and Pmt4 originated early in the evolution of opisthokonts, while Pmt1 originated later in the ancestor of fungi. Pmt1 is thus the fungi-specific member of the Pmt protein family and is the most dispensable for pathogenesis. Pmt4 is the family member with a more relevant role in virulence; in the smut fungus in particular, it is essential for appressorium formation, penetration, and hyphae extension inside plant tissues. The role of Pmt4 in appressorium formation might be controlled by O-mannosylation of Msb2, and in plant colonization the loss of Pmt4 might impact, directly or indirectly, normal activity of the protein disulfide isomerase Pdi1. By contrast, no Pmt4 substrates that could explain the penetration defects in *pmt4* mutants have been identified.

The conservation of Pmt4 in plant pathogenic fungi makes this protein, and the Pmt protein family in general, an attractive prospect for understanding the mechanisms behind plant-fungus pathosystems.

## 9. Methods

Protein search was carried out through Phmmer [84] against a local data set of selected eukaryotes (see the taxonomic tree of Figure 2a) using an *e*-value threshold of 10^−5^. We additionally performed a Phmmer search against an extensive local prokaryotic dataset (~8000 proteomes) with an *e*-value of 10^−5^. Prokaryotic sequences were reduced by a nonredundant threshold of 75% using CD-HIT [85]. Single MIR domains were re-searched using a home-made MIR protein model, built with Hmmbuild [84], using the sequence of the initial search (Figure 1 and Figure S1).

We combined all the sequences retrieved from the protein search and aligned them using Mafft-linsi [86], trimmed positions with more than 90% of gaps using trimAL [87], excluded redundant sequences up to 80% using Belvu, and removed spurious sequences by visual inspection. This dataset was used to perform the phylogeny of Figure 2a. To infer the phylogenetic profile of Figure 2b, an alternative phylogeny was carried out without a redundancy threshold and without including bacterial sequences (Figure S2).

We additionally performed a phylogenetic analysis of eukaryotic Pmt only (Figure 3b). The sequences were retrieved from the previous reconstruction using the same sequence alignment protocol. In this reconstruction, sequences from *Monosiga brevicollis* and *Fonticula alba*, suspected to provoke long-branch attraction, were excluded.

All phylogenetic trees were constructed using IQ-TREE [88] obtaining branch supports with ultrafast bootstrap (1000 replicates; [89]) and applying the automatic model selection calculated by ModelFinder and following the BIC criterion [90]. Trees were visualized and annotated using iTOL [91]. Functional domain annotation was carried out through the Pfam database [92] and transmembrane domains through the TMHMM server (http://www.cbs.dtu.dk/services/TMHMM/, accessed 21 April 2021).

## Figures and Tables

**Table 1:** Summary of the main phenotypes associated with the loss of Pmts in pathogenic fungi. V, viable; L, lethal; NT, not tested; A, severely affected; NA, not severely affected.

Fungus	Viability				Cellular and Hyphal Growth				Virulence			
	∆*pmt1*	∆*pmt2*	∆*pmt4*	∆*pmt1/4*	∆*pmt1*	∆*pmt2*	∆*pmt4*	∆*pmt1/4*	∆*pmt1*	∆*pmt2*	∆*pmt4*	∆*pmt1/4*
*Ustilago maydis* [16]	V	L	V	V	NA	L	A	A	NA	L	A	A
*Aspergillus fumigatus* [53]	V	L	V	L	NA	L	A	L	NA	L	A	L
*Aspergillus nidulans* [54]	V	V	V	NT	NA	NA	A	NT	NA	NA	NA	NT
*Cryptococcus neoformans* [55]	V	L	V	L	NA	L	A	L	A	L	A	L
*Magnaporthe oryzae* [38,56]	V	V	V	NT	NA	NA	A	NT	NA	A	A	NT
*Beauveria bassiana* [57,58]	V	L	V	NT	NA	L	NA	NT	A	L	A	NT
*Botrytis cinerea* [59]	V	V	V	NT	NA	A	A	NT	NA	A	A	NT
*Candida albicans* [60]	V	L	V	L	A	L	A	NA	NA	L	A	NA
*Fusarium oxysporum* [41]	V	L	V	V	NA	L	A	NA	NA	L	A	NA
*Penicillium digitatum* [61]	NT	V	NT	NT	NT	A	NT	NT	NT	A	NT	NT
*Metarhizium acridum* [40]	NT	NT	V	NT	NT	NT	A	NT	NT	NT	A	NT

## Data Availability

Data is contained within the article.

## Supplementary Materials

The following are available online at https://www.mdpi.com/article/10.3390/jof7050328/s1; Figure S1 (extended version of Figure 2a): Phylogeny and domain architecture of the closest homologs to Pmt proteins. The tree was artificially rooted at the split of Pmt with and without MIR domains. Question marks indicate unstable sequences in phylogenetic reconstructions. Figure S2: Phylogeny, domain architecture, and intrinsically structural disordered regions of eukaryotic Pmt proteins with no redundancy threshold. Question marks indicate unstable sequences in phylogenetic reconstructions. Sequences from *Quercus suber* and *Carpinus fangiana* likely represent genome contamination and such sequences were excluded from the analyses.

## References

[1] Varki A., Kornfeld S., Varki A., Cummings R.D., Esko J.D., Stanley P., Hart G.W., Aebi M., Darvill A.G., Kinoshita T., Packer N.H. (2015). Historical background and overview. Essentials of Glycobiology.

[2] Xu C., Ng D.T. (2015). Glycosylation-directed quality control of protein folding. Nat. Rev. Mol. Cell Biol..

[3] Varki A. (2017). Biological roles of glycans. Glycobiology.

[4] Reily C., Stewart T.J., Renfrow M.B., Novak J. (2019). Glycosylation in health and disease. Nat. Rev. Nephrol..

[5] Schjoldager K.T., Narimatsu Y., Joshi H.J., Clausen H. (2020). Global view of human protein glycosylation pathways and functions. Nat. Rev. Mol. Cell Biol..

[6] Hansen L., Lind-Thomsen A., Joshi H.J., Pedersen N.B., Have C.T., Kong Y., Wang S., Sparso T., Grarup N., Vester-Christensen M.B. (2015). A glycogene mutation map for discovery of diseases of glycosylation. Glycobiology.

[7] Freeze H.H., Eklund E.A., Ng B.G., Patterson M.C. (2015). Neurological aspects of human glycosylation disorders. Annu. Rev. Neurosci..

[8] Ng B.G., Freeze H.H. (2018). Perspectives on Glycosylation and Its Congenital Disorders. Trends Genet..

[9] Monticelli M., Ferro T., Jaeken J., Dos Reis Ferreira V., Videira P.A. (2016). Immunological aspects of congenital disorders of glycosylation (CDG): A review. J. Inherit. Metab. Dis..

[10] Pinho S.S., Reis C.A. (2015). Glycosylation in cancer: Mechanisms and clinical implications. Nat. Rev. Cancer.

[11] Corfield A.P., Berry M. (2015). Glycan variation and evolution in the eukaryotes. Trends Biochem. Sci..

[12] Calo D., Kaminski L., Eichler J. (2010). Protein glycosylation in Archaea: Sweet and extreme. Glycobiology.

[13] Nothaft H., Szymanski C.M. (2010). Protein glycosylation in bacteria: Sweeter than ever. Nat. Rev. Microbiol..

[14] Fernandez-Alvarez A., Marin-Menguiano M., Lanver D., Jimenez-Martin A., Elias-Villalobos A., Perez-Pulido A.J., Kahmann R., Ibeas J.I. (2012). Identification of O-mannosylated virulence factors in Ustilago maydis. PLoS Pathog..

[15] Schirawski J., Bohnert H.U., Steinberg G., Snetselaar K., Adamikowa L., Kahmann R. (2005). Endoplasmic reticulum glucosidase II is required for pathogenicity of Ustilago maydis. Plant Cell.

[16] Fernandez-Alvarez A., Elias-Villalobos A., Ibeas J.I. (2009). The O-mannosyltransferase PMT4 is essential for normal appressorium formation and penetration in Ustilago maydis. Plant Cell.

[17] Fernandez-Alvarez A., Elias-Villalobos A., Jimenez-Martin A., Marin-Menguiano M., Ibeas J.I. (2013). Endoplasmic reticulum glucosidases and protein quality control factors cooperate to establish biotrophy in Ustilago maydis. Plant Cell.

[18] Loibl M., Strahl S. (2013). Protein O-mannosylation: What we have learned from baker’s yeast. Biochim. Biophys. Acta.

[19] Aebi M. (2013). N-linked protein glycosylation in the ER. Biochim. Biophys. Acta.

[20] Lehle L., Strahl S., Tanner W. (2006). Protein glycosylation, conserved from yeast to man: A model organism helps elucidate congenital human diseases. Angew. Chem. Int. Ed. Engl..

[21] Spiro R.G. (2002). Protein glycosylation: Nature, distribution, enzymatic formation, and disease implications of glycopeptide bonds. Glycobiology.

[22] Schwarz F., Aebi M. (2011). Mechanisms and principles of N-linked protein glycosylation. Curr. Opin. Struct. Biol..

[23] Breitling J., Aebi M. (2013). N-linked protein glycosylation in the endoplasmic reticulum. Cold Spring Harb Perspect. Biol..

[24] Kaplan H.A., Welply J.K., Lennarz W.J. (1987). Oligosaccharyl transferase: The central enzyme in the pathway of glycoprotein assembly. Biochim. Biophys. Acta.

[25] Burda P., Aebi M. (1998). The ALG10 locus of Saccharomyces cerevisiae encodes the alpha-1,2 glucosyltransferase of the endoplasmic reticulum: The terminal glucose of the lipid-linked oligosaccharide is required for efficient N-linked glycosylation. Glycobiology.

[26] Burda P., Aebi M. (1999). The dolichol pathway of N-linked glycosylation. Biochim. Biophys. Acta.

[27] Burda P., te Heesen S., Brachat A., Wach A., Dusterhoft A., Aebi M. (1996). Stepwise assembly of the lipid-linked oligosaccharide in the endoplasmic reticulum of Saccharomyces cerevisiae: Identification of the ALG9 gene encoding a putative mannosyl transferase. Proc. Natl. Acad. Sci. USA.

[28] Knauer R., Lehle L. (1999). The oligosaccharyltransferase complex from yeast. Biochim. Biophys. Acta.

[29] Jones J., Krag S.S., Betenbaugh M.J. (2005). Controlling N-linked glycan site occupancy. Biochim. Biophys. Acta.

[30] Helenius J., Aebi M. (2002). Transmembrane movement of dolichol linked carbohydrates during N-glycoprotein biosynthesis in the endoplasmic reticulum. Semin. Cell Dev. Biol..

[31] Miwa H.E., Gerken T.A., Jamison O., Tabak L.A. (2010). Isoform-specific O-glycosylation of osteopontin and bone sialoprotein by polypeptide N-acetylgalactosaminyltransferase-1. J. Biol. Chem..

[32] Koomey M. (2019). O-linked protein glycosylation in bacteria: Snapshots and current perspectives. Curr. Opin. Struct. Biol..

[33] West C.M., Kim H.W. (2019). Nucleocytoplasmic O-glycosylation in protists. Curr. Opin. Struct. Biol..

[34] Lommel M., Strahl S. (2009). Protein O-mannosylation: Conserved from bacteria to humans. Glycobiology.

[35] Goto M. (2007). Protein O-glycosylation in fungi: Diverse structures and multiple functions. Biosci. Biotechnol. Biochem..

[36] Neubert P., Strahl S. (2016). Protein O-mannosylation in the early secretory pathway. Curr. Opin. Cell Biol..

[37] Marin-Menguiano M., Moreno-Sanchez I., Barrales R.R., Fernandez-Alvarez A., Ibeas J.I. (2019). N-glycosylation of the protein disulfide isomerase Pdi1 ensures full Ustilago maydis virulence. PLoS Pathog..

[38] Pan Y., Pan R., Tan L., Zhang Z., Guo M. (2019). Pleiotropic roles of O-mannosyltransferase MoPmt4 in development and pathogenicity of Magnaporthe oryzae. Curr. Genet..

[39] Zhao G., Xu Y., Ouyang H., Luo Y., Sun S., Wang Z., Yang J., Jin C. (2020). Protein O-mannosylation affects protein secretion, cell wall integrity and morphogenesis in Trichoderma reesei. Fungal Genet. Biol..

[40] Zhao T., Tian H., Xia Y., Jin K. (2019). MaPmt4, a protein O-mannosyltransferase, contributes to cell wall integrity, stress tolerance and virulence in Metarhizium acridum. Curr. Genet..

[41] Xu Y., Zhou H., Zhao G., Yang J., Luo Y., Sun S., Wang Z., Li S., Jin C. (2020). Genetical and O-glycoproteomic analyses reveal the roles of three protein O-mannosyltransferases in phytopathogen Fusarium oxysporum f.sp. cucumerinum. Fungal Genet. Biol..

[42] VanderVen B.C., Harder J.D., Crick D.C., Belisle J.T. (2005). Export-mediated assembly of mycobacterial glycoproteins parallels eukaryotic pathways. Science.

[43] Matei A., Doehlemann G. (2016). Cell biology of corn smut disease-Ustilago maydis as a model for biotrophic interactions. Curr. Opin. Microbiol..

[44] Mendoza-Mendoza A., Berndt P., Djamei A., Weise C., Linne U., Marahiel M., Vranes M., Kamper J., Kahmann R. (2009). Physical-chemical plant-derived signals induce differentiation in Ustilago maydis. Mol. Microbiol..

[45] Lanver D., Tollot M., Schweizer G., Lo Presti L., Reissmann S., Ma L.S., Schuster M., Tanaka S., Liang L., Ludwig N. (2017). Ustilago maydis effectors and their impact on virulence. Nat. Rev. Microbiol..

[46] Ponting C.P. (2000). Novel repeats in ryanodine and IP3 receptors and protein O-mannosyltransferases. Trends Biochem. Sci..

[47] Albuquerque-Wendt A., Hutte H.J., Buettner F.F.R., Routier F.H., Bakker H. (2019). Membrane Topological Model of Glycosyltransferases of the GT-C Superfamily. Int. J. Mol. Sci..

[48] Bai L., Kovach A., You Q., Kenny A., Li H. (2019). Structure of the eukaryotic protein O-mannosyltransferase Pmt1-Pmt2 complex. Nat. Struct. Mol. Biol..

[49] Chiapparino A., Grbavac A., Jonker H.R., Hackmann Y., Mortensen S., Zatorska E., Schott A., Stier G., Saxena K., Wild K. (2020). Functional implications of MIR domains in protein O-mannosylation. Elife.

[50] Girrbach V., Strahl S. (2003). Members of the evolutionarily conserved PMT family of protein O-mannosyltransferases form distinct protein complexes among themselves. J. Biol. Chem..

[51] Akasaka-Manya K., Manya H., Nakajima A., Kawakita M., Endo T. (2006). Physical and functional association of human protein O-mannosyltransferases 1 and 2. J. Biol. Chem..

[52] Kriangkripipat T., Momany M. (2014). Aspergillus nidulans Pmts form heterodimers in all pairwise combinations. FEBS Open Bio.

[53] Mouyna I., Kniemeyer O., Jank T., Loussert C., Mellado E., Aimanianda V., Beauvais A., Wartenberg D., Sarfati J., Bayry J. (2010). Members of protein O-mannosyltransferase family in Aspergillus fumigatus differentially affect growth, morphogenesis and viability. Mol. Microbiol..

[54] Kriangkripipat T., Momany M. (2009). Aspergillus nidulans protein O-mannosyltransferases play roles in cell wall integrity and developmental patterning. Eukaryot. Cell.

[55] Olson G.M., Fox D.S., Wang P., Alspaugh J.A., Buchanan K.L. (2007). Role of protein O-mannosyltransferase Pmt4 in the morphogenesis and virulence of Cryptococcus neoformans. Eukaryot. Cell.

[56] Guo M., Tan L., Nie X., Zhu X., Pan Y., Gao Z. (2016). The Pmt2p-Mediated Protein O-Mannosylation Is Required for Morphogenesis, Adhesive Properties, Cell Wall Integrity and Full Virulence of Magnaporthe oryzae. Front. Microbiol..

[57] Wang J.J., Qiu L., Chu Z.J., Ying S.H., Feng M.G. (2014). The connection of protein O-mannosyltransferase family to the biocontrol potential of Beauveria bassiana, a fungal entomopathogen. Glycobiology.

[58] He Z., Luo L., Keyhani N.O., Yu X., Ying S., Zhang Y. (2017). The C-terminal MIR-containing region in the Pmt1 O-mannosyltransferase restrains sporulation and is dispensable for virulence in Beauveria bassiana. Appl. Microbiol. Biotechnol..

[59] Gonzalez M., Brito N., Frias M., Gonzalez C. (2013). Botrytis cinerea protein O-mannosyltransferases play critical roles in morphogenesis, growth, and virulence. PLoS ONE.

[60] Prill S.K., Klinkert B., Timpel C., Gale C.A., Schroppel K., Ernst J.F. (2005). PMT family of Candida albicans: Five protein mannosyltransferase isoforms affect growth, morphogenesis and antifungal resistance. Mol. Microbiol..

[61] Harries E., Gandia M., Carmona L., Marcos J.F. (2015). The Penicillium digitatum protein O-mannosyltransferase Pmt2 is required for cell wall integrity, conidiogenesis, virulence and sensitivity to the antifungal peptide PAF26. Mol. Plant. Pathol..

[62] Mahne M., Tauch A., Puhler A., Kalinowski J. (2006). The Corynebacterium glutamicum gene pmt encoding a glycosyltransferase related to eukaryotic protein-O-mannosyltransferases is essential for glycosylation of the resuscitation promoting factor (Rpf2) and other secreted proteins. FEMS Microbiol. Lett..

[63] Liu C.F., Tonini L., Malaga W., Beau M., Stella A., Bouyssie D., Jackson M.C., Nigou J., Puzo G., Guilhot C. (2013). Bacterial protein-O-mannosylating enzyme is crucial for virulence of Mycobacterium tuberculosis. Proc. Natl. Acad. Sci. USA.

[64] Wehmeier S., Varghese A.S., Gurcha S.S., Tissot B., Panico M., Hitchen P., Morris H.R., Besra G.S., Dell A., Smith M.C. (2009). Glycosylation of the phosphate binding protein, PstS, in Streptomyces coelicolor by a pathway that resembles protein O-mannosylation in eukaryotes. Mol. Microbiol..

[65] Haines N., Seabrooke S., Stewart B.A. (2007). Dystroglycan and protein O-mannosyltransferases 1 and 2 are required to maintain integrity of Drosophila larval muscles. Mol. Biol. Cell.

[66] Martin-Blanco E., Garcia-Bellido A. (1996). Mutations in the rotated abdomen locus affect muscle development and reveal an intrinsic asymmetry in Drosophila. Proc. Natl. Acad. Sci. USA.

[67] Baker R., Nakamura N., Chandel I., Howell B., Lyalin D., Panin V.M. (2018). Protein O-Mannosyltransferases Affect Sensory Axon Wiring and Dynamic Chirality of Body Posture in the Drosophila Embryo. J. Neurosci..

[68] Willer T., Prados B., Falcon-Perez J.M., Renner-Muller I., Przemeck G.K., Lommel M., Coloma A., Valero M.C., de Angelis M.H., Tanner W. (2004). Targeted disruption of the Walker-Warburg syndrome gene Pomt1 in mouse results in embryonic lethality. Proc. Natl. Acad. Sci. USA.

[69] Beltran-Valero de Bernabe D., Currier S., Steinbrecher A., Celli J., van Beusekom E., van der Zwaag B., Kayserili H., Merlini L., Chitayat D., Dobyns W.B. (2002). Mutations in the O-mannosyltransferase gene POMT1 give rise to the severe neuronal migration disorder Walker-Warburg syndrome. Am. J. Hum. Genet..

[70] Akasaka-Manya K., Manya H., Endo T. (2004). Mutations of the POMT1 gene found in patients with Walker-Warburg syndrome lead to a defect of protein O-mannosylation. Biochem. Biophys. Res. Commun..

[71] Gentzsch M., Tanner W. (1996). The PMT gene family: Protein O-glycosylation in Saccharomyces cerevisiae is vital. EMBO J..

[72] Strahl-Bolsinger S., Scheinost A. (1999). Transmembrane topology of pmt1p, a member of an evolutionarily conserved family of protein O-mannosyltransferases. J. Biol. Chem..

[73] Nett J.H., Cook W.J., Chen M.T., Davidson R.C., Bobrowicz P., Kett W., Brevnova E., Potgieter T.I., Mellon M.T., Prinz B. (2013). Characterization of the Pichia pastoris protein-O-mannosyltransferase gene family. PLoS ONE.

[74] Willer T., Brandl M., Sipiczki M., Strahl S. (2005). Protein O-mannosylation is crucial for cell wall integrity, septation and viability in fission yeast. Mol. Microbiol..

[75] Willger S.D., Ernst J.F., Alspaugh J.A., Lengeler K.B. (2009). Characterization of the PMT gene family in Cryptococcus neoformans. PLoS ONE.

[76] Liu C., Talbot N.J., Chen X.L. (2021). Protein glycosylation during infection by plant pathogenic fungi. New Phytol..

[77] Fernandez-Alvarez A., Elias-Villalobos A., Ibeas J.I. (2010). The requirement for protein O-mannosylation for Ustilago maydis virulence seems to be linked to intrinsic aspects of the infection process rather than an altered plant response. Plant. Signal. Behav..

[78] Hutzler J., Schmid M., Bernard T., Henrissat B., Strahl S. (2007). Membrane association is a determinant for substrate recognition by PMT4 protein O-mannosyltransferases. Proc. Natl. Acad. Sci. USA.

[79] Lanver D., Mendoza-Mendoza A., Brachmann A., Kahmann R. (2010). Sho1 and Msb2-related proteins regulate appressorium development in the smut fungus Ustilago maydis. Plant. Cell.

[80] Liu W., Zhou X., Li G., Li L., Kong L., Wang C., Zhang H., Xu J.R. (2011). Multiple plant surface signals are sensed by different mechanisms in the rice blast fungus for appressorium formation. PLoS Pathog..

[81] Lanver D., Berndt P., Tollot M., Naik V., Vranes M., Warmann T., Munch K., Rossel N., Kahmann R. (2014). Plant surface cues prime Ustilago maydis for biotrophic development. PLoS Pathog..

[82] Doehlemann G., Reissmann S., Assmann D., Fleckenstein M., Kahmann R. (2011). Two linked genes encoding a secreted effector and a membrane protein are essential for Ustilago maydis-induced tumour formation. Mol. Microbiol..

[83] Djamei A., Schipper K., Rabe F., Ghosh A., Vincon V., Kahnt J., Osorio S., Tohge T., Fernie A.R., Feussner I. (2011). Metabolic priming by a secreted fungal effector. Nature.

[84] Potter S.C., Luciani A., Eddy S.R., Park Y., Lopez R., Finn R.D. (2018). HMMER web server: 2018 update. Nucleic Acids Res..

[85] Fu L., Niu B., Zhu Z., Wu S., Li W. (2012). CD-HIT: Accelerated for clustering the next-generation sequencing data. Bioinformatics.

[86] Katoh K., Standley D.M. (2013). MAFFT multiple sequence alignment software version 7: Improvements in performance and usability. Mol. Biol. Evol..

[87] Capella-Gutierrez S., Silla-Martinez J.M., Gabaldon T. (2009). trimAl: A tool for automated alignment trimming in large-scale phylogenetic analyses. Bioinformatics.

[88] Nguyen L.T., Schmidt H.A., von Haeseler A., Minh B.Q. (2015). IQ-TREE: A fast and effective stochastic algorithm for estimating maximum-likelihood phylogenies. Mol. Biol. Evol..

[89] Hoang D.T., Chernomor O., von Haeseler A., Minh B.Q., Vinh L.S. (2018). UFBoot2: Improving the Ultrafast Bootstrap Approximation. Mol. Biol. Evol..

[90] Kalyaanamoorthy S., Minh B.Q., Wong T.K.F., von Haeseler A., Jermiin L.S. (2017). ModelFinder: Fast model selection for accurate phylogenetic estimates. Nat. Methods.

[91] Letunic I., Bork P. (2019). Interactive Tree Of Life (iTOL) v4: Recent updates and new developments. Nucleic Acids Res..

[92] Finn R.D., Bateman A., Clements J., Coggill P., Eberhardt R.Y., Eddy S.R., Heger A., Hetherington K., Holm L., Mistry J. (2014). Pfam: The protein families database. Nucleic Acids Res..

