# Supplementary figures and images for: Structural, Evolutionary, and Functional Analysis of the Protein O-Mannosyltransferase Family in Pathogenic Fungi
Source: J Fungi (Basel). 2021 Apr 23;7(5):328. doi: 10.3390/jof7050328 (PMC8147084; doi:10.3390/jof7050328)

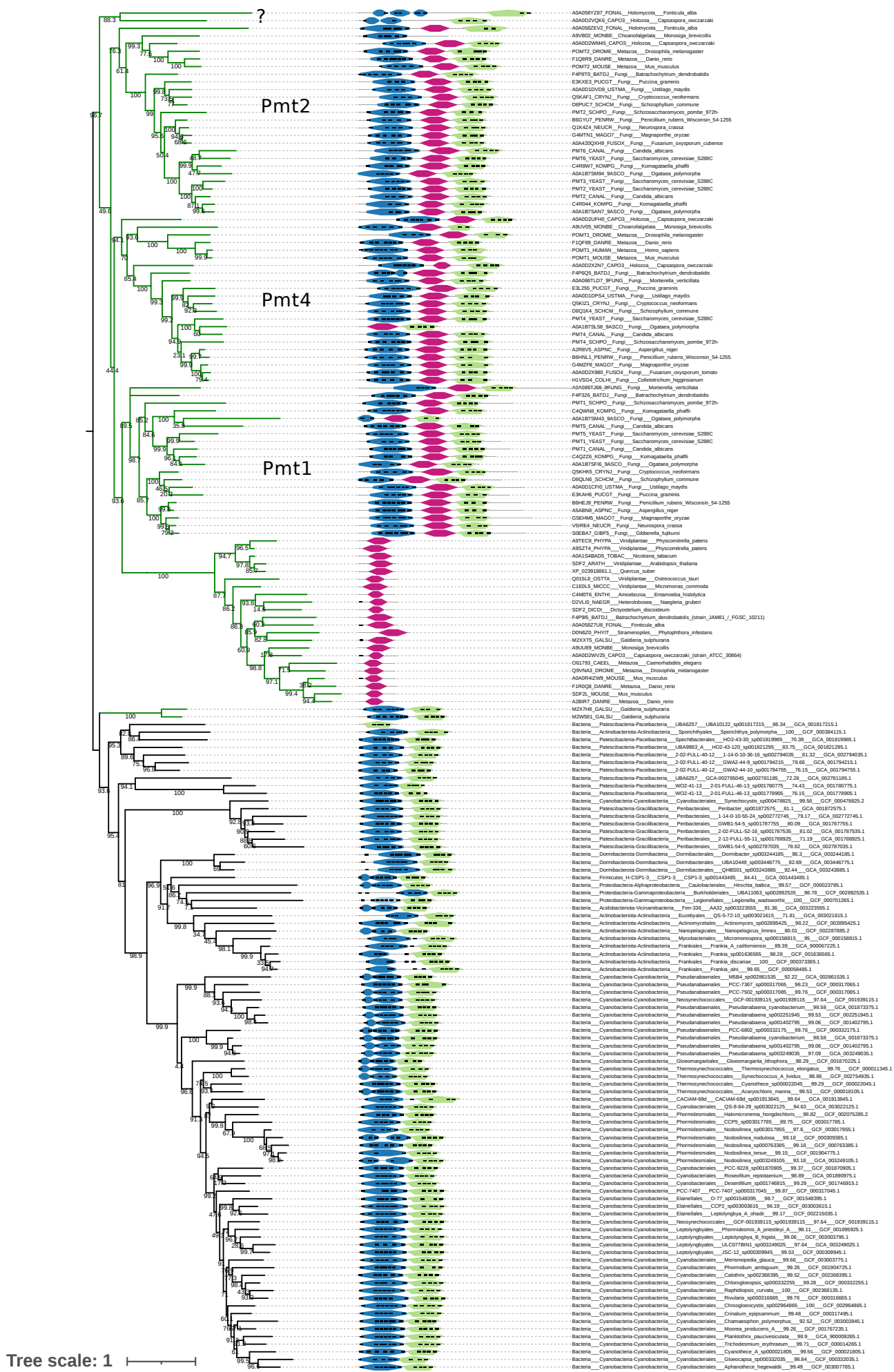

Supplement: Supplementary file 1 [file jof-07-00328-s001.zip › Supplementary_Figures/Supplementary_1[1].pdf]

Tree scale: 1

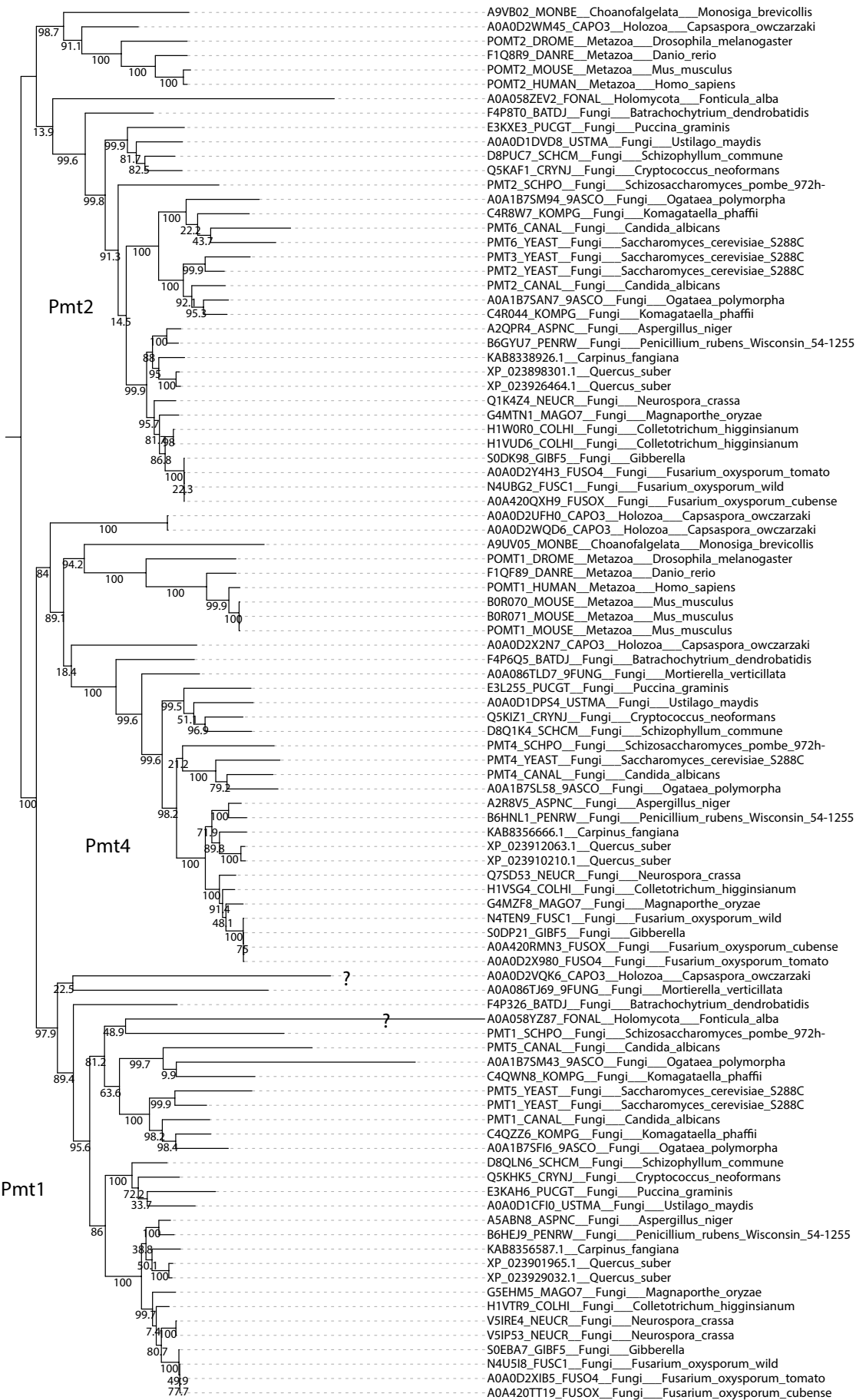

Structural disorder (IUPred)

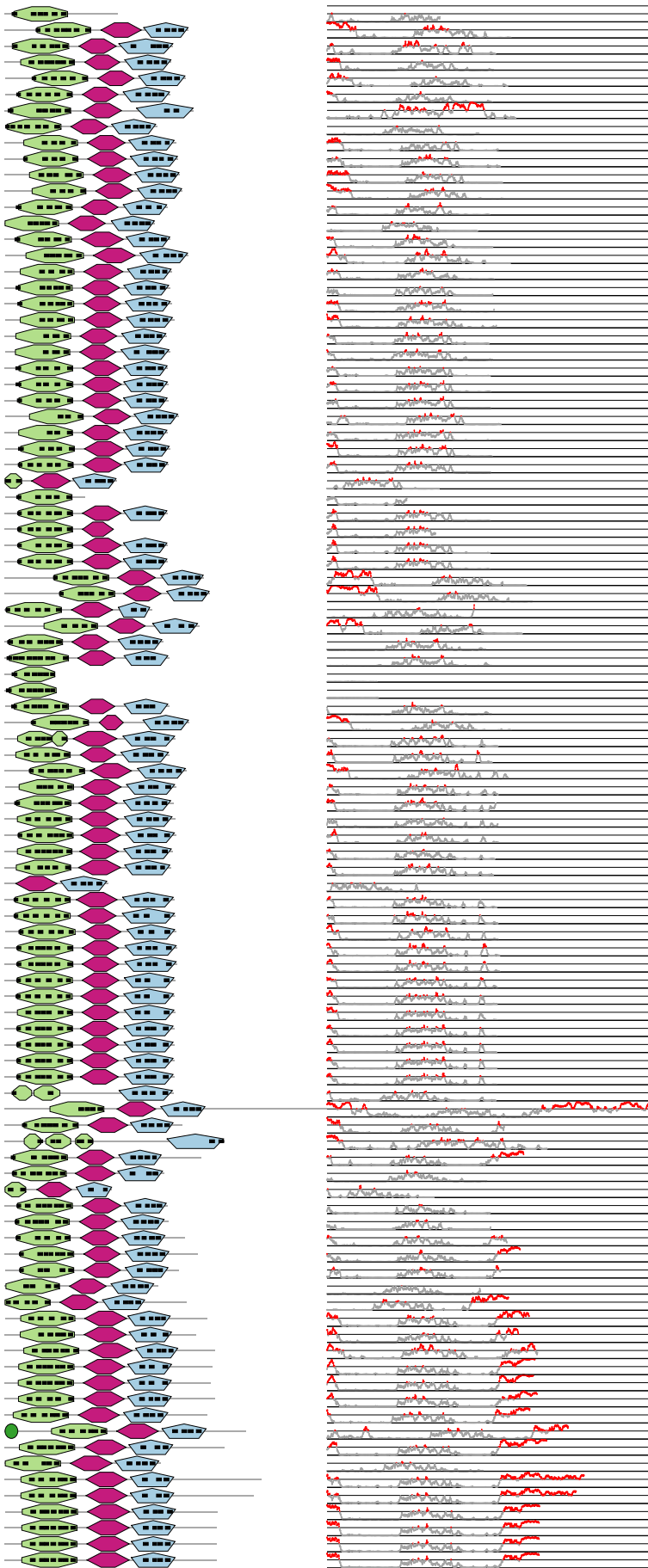

Supplement: Supplementary file 1 [file jof-07-00328-s001.zip › Supplementary_Figures/Supplementary_2[1].pdf]
